# The role of tenofovir-based HIV pre-exposure prophylaxis in preventing HBV infection among men who have sex with men: insights from China

**DOI:** 10.1186/s40249-025-01305-9

**Published:** 2025-04-27

**Authors:** Zhen-Hao Wu, Yan-Yan Zhu, Xiao-Jie Huang, Shuo Chen, Zhen-Xing Chu, Hui Wang, Yao-Kai Chen, Yong-Jun Jiang, Hong Shang, Qing-Hai Hu

**Affiliations:** 1 State Key Laboratory for Diagnosis and Treatment of Infectious Diseases, NHC Key Laboratory of AIDS Prevention and Treatment, National Clinical Research Center for Laboratory Medicine, The First Hospital of China Medical University, China Medical University, Shenyang, 110001 China; 2 Key Laboratory of AIDS Immunology, Chinese Academy of Medical Sciences, Shenyang, 110001 China; 3 Key Laboratory of AIDS Immunology of Liaoning Province, Shenyang, 110001 China; 4 Collaborative Innovation Center for Diagnosis and Treatment of Infectious Diseases, 79 Qingchun Street, Hangzhou, 310003 China; 5 Center for Infectious Diseases, Beijing Youan Hospital, Capital Medical University, Beijing, China; 6 Department of Infectious Diseases, National Clinical Center for Infectious Diseases, Third People’s Hospital of Shenzhen (Second Affiliated Hospital of Southern University of Science and Technology), Shenzhen, China; 7 Chongqing Public Health Medical Center, Chongqing, China

**Keywords:** Pre-exposure prophylaxis, Hepatitis B virus, Hepatitis C virus, Men who have sex with men, Incidence, Bayesian Poisson regression

## Abstract

**Background:**

Oral emtricitabine-tenofovir disoproxil fumarate (F/TDF) for HIV pre-exposure prophylaxis (PrEP) demonstrates dual potential through antiviral activity against hepatitis B virus (HBV). While F/TDF lacks activity against hepatitis C virus (HCV), the use of F/TDF for HIV PrEP may elevate HCV risk through risk compensation. This study aims to investigate HBV/HCV incidence among men who have sex with men (MSM) using F/TDF-based HIV PrEP, addressing evidence gaps in low- and middle-income countries.

**Methods:**

We conducted a secondary analysis of the China Real-World Oral Intake of PrEP (CROPrEP) study, a multicenter prospective cohort of MSM (F/TDF users/non-users) from Beijing, Shenyang, Shenzhen, and Chongqing. Participants underwent HBV/HCV testing at baseline and at the 12-month follow-up. Only HBV-susceptible (hepatitis B surface antigen-negative, hepatitis B surface and core antibody-negative) MSM were included in the secondary analysis, to calculate HBV incidence. The primary outcomes were HBV/HCV incidence rates at the 12-month follow-up. Bayesian Poisson regression identified HBV/HCV infection risk factors.

**Results:**

The CROPrEP cohort prospectively recruited 1023 F/TDF users and 507 F/TDF non-users at baseline. This secondary analysis included 259 F/TDF users and 120 non-users identified as HBV-susceptible at baseline. At the 12-month of follow-up, no incident HBV infections occurred in the F/TDF users group, and only one incident HBV infection occurred in the F/TDF non-users group. The incidence of new HBV infections was 0.00/100 person-years (PY) [95% confidence interval (*CI*): 0.00**–**1.32] among HBV-susceptible F/TDF users and 0.77/100 PY (95% *CI:* 0.02–4.20) among HBV-susceptible F/TDF non-users. HBV incidence was reduced with F/TDF compared with no F/TDF [adjusted incidence rate ratio (aIRR): 0.00; 95% *CI:* 0.00–0.00]. HCV incidence among F/TDF users and non-users was 0.31/100 PY (95% *CI:* 0.06–0.90) and 0.00/100 PY (95% *CI:* 0.00–0.74) after 12 months, respectively. HCV incidence was lower in F/TDF non-users than in F/TDF users (aIRR: 0.00; 95% *CI:* 0.00–0.25).

**Conclusions:**

This study suggests a potential benefit in reducing HBV incidence among MSM using F/TDF as HIV PrEP, highlighting the potential for integrated prevention strategies addressing both HIV and HBV risks in PrEP programmes.

*Trial registration*: ChiCTR, ChiCTR-IIN-17013762. Registered 8 December 2017, https://www.chictr.org.cn/showproj.html?proj=22916.

**Graphical Abstract:**

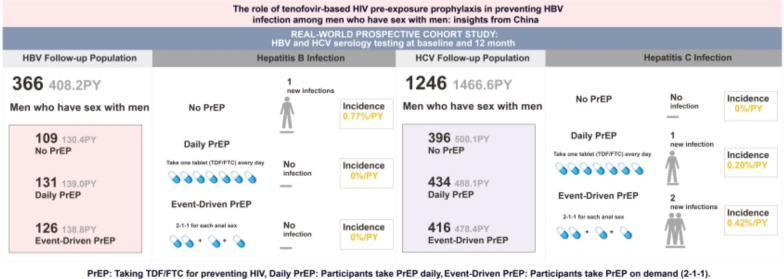

**Supplementary Information:**

The online version contains supplementary material available at 10.1186/s40249-025-01305-9.

## Background

Globally, men who have sex with men (MSM) are at high risk for hepatitis B virus (HBV) and hepatitis C virus (HCV) through sexual contact [1]. Vaccination is the key strategy for preventing HBV in this population, but implementation remains challenging [2]. In the European Union, MSM are classified as a high-risk group for HBV, with prevalence between 0.2% and 3.4% [3, 4]. In China, meta-analyses indicate a higher HBV prevalence among MSM, ranging from 7.9% to 8.9% [5, 6]. The risk of HCV transmission is elevated among individuals at risk for HIV acquisition and people living with HIV, with a global prevalence of 3.4% among MSM [7]. In China, the seroprevalence of the HCV antibody (anti-HCV) among MSM is approximately 0.84% [8]. The World Health Organization aims to eliminate hepatitis as a public health threat by 2030 [9], emphasising the importance of addressing HBV and HCV in MSM. However, research on HBV and HCV infections among HIV-negative MSM, particularly in low-income and middle-income countries (LMICs), including China, remains limited.

Daily or event-driven oral emtricitabine-tenofovir disoproxil fumarate (F/TDF) is an effective HIV pre-exposure prophylaxis (PrEP) strategy for MSM [10–13]. However, HIV PrEP use may increase the risk of sexually transmitted infections (STIs), possibly due to lower condom use or more sexual partners [14, 15]. Studies indicate that HIV-negative MSM on PrEP have a higher risk of HCV infection than PrEP non-users [16, 17]. A meta-analysis conducted in 2020 reported a higher HCV incidence rate in PrEP users [14.8/1000 person-years (PY)] than in PrEP non-users (0.12/1000 PY) [7]. The increasing recognition that individuals on antiretroviral therapy with fully suppressed viral loads cannot transmit HIV may further reduce condom use among MSM with differing HIV statuses [18], potentially increasing HCV transmission risk through overlapping sexual networks [17].

Studies on F/TDF in HIV-infected individuals suggest that tenofovir-based PrEP might protect against both HIV and HBV [19, 20] because tenofovir has activity against HBV and is used to treat people living with HBV [21]. To date, only one study from Japan has demonstrated the efficacy of tenofovir-based PrEP in preventing HBV among MSM in real-world settings [21]. However, more data from diverse settings are needed to validate these preliminary results.

Most research on HCV and HBV among MSM on PrEP has been conducted in high-income countries [22], with less understanding in LMICs. Although prior studies have documented HBV vaccination rates among Chinese MSM [23, 24], none have combined serological confirmation of immunity with a focus on PrEP users, a population uniquely positioned to benefit from integrated prevention services. This study aims to investigate HBV and HCV incidence among HIV-negative MSM who use F/TDF in China, and to describe HBV vaccination status among MSM on HIV PrEP.

## Methods

### Study design and population

The China Real-World Oral Intake of PrEP (CROPrEP) study was a multicenter, real-world study conducted from 11 December 2018 to 30 November 2020 in Beijing, Shenyang, Shenzhen and Chongqing. Eligible F/TDF users were men aged 18 to 65 years who were HIV-seronegative, had oral and/or anal intercourse with men in their lifetime, and reported at least one sexual risk criterion in the past six months [ie, condomless anal intercourse (CAI) with men,  ≥ 2 male sexual partners, history of STI diagnosis, or use of post-exposure prophylaxis]. The exclusion criteria consisted of (1) HIV infection, (2) serious chronic diseases (e.g., metabolic diseases or neurological and psychiatric disorders), (3) liver and kidney dysfunction [i.e., creatinine clearance (estimated glomerular filtration rate)  < 60 ml/min or severe liver or kidney dysfunction], (4) hepatitis B surface antigen (HBsAg) positivity, (5) the use of antiretrovirals, interferons, or interleukins. F/TDF non-users were subject to the same eligibility criteria and exclusion criterion 1 (baseline HIV negativity) as F/TDF users; exclusion criteria 2–5 (e.g., renal impairment, HBsAg positivity) applied only to F/TDF users. All participants who tested HIV-positive during follow-up were referred to care and excluded from further study procedures.

Participants were assigned to the F/TDF users group and non-users groups depending on their decision whether to start using F/TDF at the baseline of the CROPrEP study. For the daily F/TDF group, participants took one tablet (F/TDF) per day. For the event-driven F/TDF group, two tablets were orally administered 2 to 24 h before each anal intercourse, followed by one tablet each at 24 and 48 h after the initial dose. Within the 12-month follow-up, participants in the F/TDF users groups could switch between daily and event-driven groups. All participants provided written informed consent and completed questionnaires during the study. Follow-up visits and blood sample collections were conducted at 0, 1, 3, 6, 9 and 12 months to facilitate HIV and related laboratory testing. The remaining samples were aliquoted and stored at − 80 °C for further testing.

According to CROPrEP protocol [25], HBsAg testing was performed for both F/TDF users and non-users at baseline, with repeated testing conducted at the 12-month follow-up. Following baseline testing, all participants received standardized post-test counselling and were recommended to initiate self-funded HBV vaccination. While systematic collection of HBV vaccination histories was not implemented during follow-up, supplementary telephone interviews were conducted for individuals with incident HBV seroconversion to retrospectively ascertain vaccination status.

This secondary analysis aimed to assess the 12-month HBV incidence rates among daily and event-driven F/TDF users compared to F/TDF non-users. Participants with available baseline specimens underwent additional serological testing for antibody to hepatitis B surface antigen (anti-HBs) and antibody to hepatitis B core antigen (anti-HBc). Eligibility for HBV incidence calculation of this secondary analysis required comprehensive serological testing at baseline: HBsAg-negative, anti-HBs- and anti-HBc-negative. Anti-HCV testing was conducted for both F/TDF users and F/TDF non-users using stored serum samples obtained at baseline and the 12-month follow-up visit.

### Infection outcomes and incidence rate calculation

Incident HBV infection was defined as HBV-susceptible (negative HBsAg, anti-HBs and anti-HBc) at baseline and HBsAg-positive at the 12-month follow-up. Similarly, incident HCV infection was defined as negative anti-HCV at baseline and positive at the 12-month follow-up. HBV incidence rate was calculated based on susceptible individuals.

### Detection and classification of HBV and HCV serological markers

An enzyme-linked immunosorbent assay (ELISA) kit (Wantai, Beijing, China) was used to detect HBsAg, anti-HBc, and anti-HBs at enrolment. Susceptibility to HBV infection was defined as the absence of all three serologic markers (HBsAg−, anti-HBc−, anti-HBs−). Vaccine-associated immunity to HBV was defined as the presence of only anti-HBs (HBsAg−, anti-HBc−, anti-HBs+). Current HBV infection was defined by the presence of HBsAg confirmed with anti-HBc (HBsAg+, anti-HBc+, anti-HBs−). Immunity due to natural infection (past infection) was defined as the presence of anti-HBc and anti-HBs without HBsAg (HBsAg−, anti-HBc+, anti-HBs+). At the 12-month follow-up, only HBsAg was tested using the same ELISA method.

Anti-HCV was detected with an ELISA assay (Wantai, Beijing, China) at enrolment and the 12-month follow-up. Confirmation of positive results was mandated, and laboratory protocols were strictly followed. Participants with a positive HBsAg or anti-HCV result were referred for confirmatory testing and linkage-to-care services.

### Data collection

This secondary analysis of the CROPrEP project did not have a predefined sample size for HBV and HCV incidence. Only HBV-susceptible (HBsAg−, anti-HBc−, anti-HBs−) MSM were included in the secondary analysis to calculate HBV incidence. The “Jinshuju” online form tool (https://jinshuju.net/home) was used to collect self-reported questionnaire data. The questionnaire included measures on: (1) demographics such as age, marital status, education, and monthly income; (2) sexual behaviours in the past three months such as the number of regular or casual male sexual partners and experiences of group sex and CAI; and (3) history of HBV vaccination in the past 10 years (assessed by self-report “yes/no/cannot remember”).

We developed a model to impute missing values for variables by using those without missing data, including HBV and HCV infections, through multiple chained equation iterations, yielding complete datasets. We then evaluated the imputation results for reliability and selected the best dataset.

### Statistical analysis

Results are presented as median and interquartile range (IQR) for continuous variables and as frequency and percentage for categorical variables. Age differences were analysed using the Mann–Whitney *U* test and categorical variables were compared using the chi-square test. We calculated the 95% confidence intervals (*CI*s) for the incidence per 100 PY using the binomial distribution. Bayesian Poisson regression was used to estimate incidence rate ratios of HBV and HCV infections and 95% *CI*s. In multivariate regression models, F/TDF use was analysed as the primary exposure variable. Adjusted incidence rate ratios (aIRR) were estimated after controlling for covariates including age, CAI in the past three months, monthly frequency of sexual intercourse in the past three months, number of casual male sexual partners in the past three months, and education level. Statistical significance was determined with a two-tailed *α* of 0.05, and all statistical analyses were performed using the software SPSS 29.0 (IBM Inc., Armonk, NY, USA) and R 4.3.1 (Lucent Technologies, Jasmine Mountain, USA).

## Results

### Baseline characteristics of study participants

**Fig. 1:**
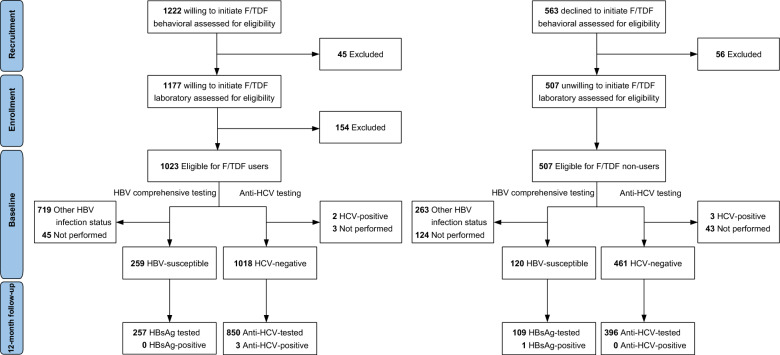
Study Profile. HBV-susceptible: HBsAg−, anti-HBc−, anti-HBs−. *HBV* hepatitis B virus, *HCV* hepatitis C virus, *F/TDF* emtricitabine-tenofovir disoproxil fumarate, *HBsAg* hepatitis B surface antigen, *Anti-HBc* antibody to hepatitis B core antigen, *Anti-HBs* antibody to hepatitis B surface antigen, *Anti-HCV* HCV antibody

Figure 1 illustrates the recruitment process. At baseline, 978 F/TDF users underwent HBV comprehensive testing. Of them, 525 (53.7%) had serologic evidence of vaccination, while 259 (26.5%) were susceptible to HBV infection (Supplementary Material Table S1). The median age of the 259 HBV-susceptible F/TDF users was 33 years (IQR: 28–38 years) at baseline. Most F/TDF users resided in Beijing (42.1%) and reported having more than two casual partners (48.3%). Among HBV-susceptible F/TDF users, 115 (44.4%) self-reported having received HBV vaccination in the past 10 years (Table 1).

**Table 1:** Baseline differences between F/TDF users and F/TDF non-users [MSM who were HBV-susceptible (HBsAg−, anti-HBs−, anti-HBc−), *n* = 379]

Characteristic	F/TDF non-users^a^ (*n* = 120)	F/TDF users^a^ (*n* = 259)	*P* value^b^
Age, median (IQR), years	38 (31–44)	33 (28–38)	** < 0.001**
Age, years			** < 0.001**
18–24	9 (7.5)	14 (5.4)	
25–34	33 (27.5)	144 (55.6)	
35–44	48 (40.0)	73 (28.2)	
> 44	30 (25.0)	28 (10.8)	
Survey site			0.879
Shenyang	47 (39.2)	109 (42.1)	
Beijing	39 (32.5)	75 (29.0)	
Shenzhen	7 (5.8)	18 (6.9)	
Chongqing	27 (22.5)	57 (22.0)	
Educational level			** < 0.001**
High school or less	59 (49.2)	59 (22.8)	
College or greater	61 (50.8)	200 (77.2)	
Monthly income, USD			0.455
< 619	42 (35.0)	101 (39.0)	
≥ 619	78 (65.0)	158 (61.0)	
Gender identity			**0.034**
Male	111 (92.5)	253 (97.7)	
Female	9 (7.5)	6 (2.3)	
Marital status			**0.001**
Single	57 (47.5)	143 (55.2)	
Married/cohabitation with a woman	22 (18.3)	16 (6.2)	
Cohabitation with a man	36 (30.0)	95 (36.7)	
Separated, divorced, or widowed	5 (4.2)	5 (1.9)	
Sexual orientation			0.312
Homosexual	88 (73.4)	193 (74.5)	
Bisexual	25 (20.8)	59 (22.8)	
Other	7 (5.8)	7 (2.7)	
Received HBV vaccination in the past 10 years			0.379
No	16 (13.3)	48 (18.5)	
Yes	53 (44.2)	115 (44.4)	
Can not remember	51 (42.5)	96 (37.1)	
Monthly frequency of sexual intercourse in the past three months			** < 0.001**
None	60 (50.0)	32 (12.4)	
1–3	33 (27.5)	135 (52.1)	
> 3	27 (22.5)	92 (35.5)	
Sexual role with men in the past three months			0.487
Top	46 (38.3)	86 (33.2)	
Bottom	22 (18.3)	64 (24.7)	
Versatile	49 (40.9)	100 (38.6)	
Oral	3 (2.5)	9 (3.5)	
Number of regular male sexual partner in the past three months			0.344
None	57 (47.5)	105 (40.5)	
1–2	41 (34.2)	92 (35.6)	
> 2	22 (18.3)	62 (23.9)	
Number of casual male sexual partner in the past three months			**0.024**
None	51 (42.5)	85 (32.8)	
1–2	29 (24.2)	49 (18.9)	
> 2	40 (33.3)	125 (48.3)	
Group sex in the past three months			**0.032**
No	107 (89.2)	208 (80.3)	
Yes	13 (10.8)	51 (19.7)	
CAI in the past three months			0.132
No	59 (49.2)	106 (40.9)	
Yes	61 (50.8)	153 (59.1)	

Statistical significance was defined as *P* < 0.05, with significant values highlighted in bold

*F/TDF* emtricitabine-tenofovir disoproxil fumarate, *IQR* interquartile range, *HBsAg* hepatitis B surface antigen, *anti-HBc* antibody to hepatitis B core antigen, *anti-HBs* antibody to hepatitis B surface antigen, *CAI* condomless anal intercourse, *MSM* men who have sex with men, *USD* the United States Dollar

^a^For this secondary analysis, participants were assigned to the F/TDF users group and non-users groups depending on their decision whether to start using F/TDF at the baseline of the CROPrEP study

^b^Chi-squared or Fisher exact test was applied for categorical variables

Conversely, 383 F/TDF non-users underwent HBV comprehensive testing. Of them, 130 (33.9%) had serologic evidence of vaccination, while 120 (31.3%) were susceptible to HBV infection (Supplementary Material Table S2). The median age of the 120 HBV-susceptible F/TDF non-users was 38 years (IQR: 31–44 years) at baseline, with the majority residing in Shenyang (39.2%). Only 53 HBV-susceptible individuals (44.2%) self-reported having received HBV vaccination in the past 10 years (Table 1).

At baseline, 1020 F/TDF users and 464 F/TDF non-users underwent anti-HCV testing.

### HBV and HCV incidence

**Fig. 2:**
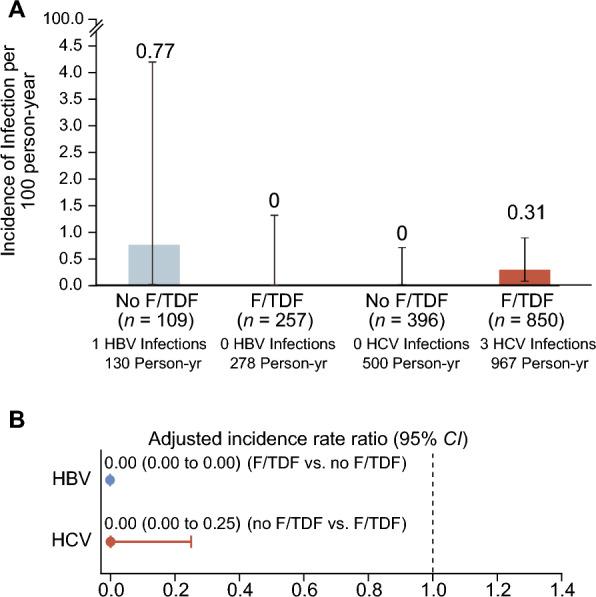
Incidence of HBV and HCV Infection. **A** HBV and HCV incidence rate in F/TDF users and F/TDF non-users. **B** Incidence rate ratio comparing HBV and HCV incidence in F/TDF users and F/TDF non-users. In Panel A, the I bars indicate 95% confidence intervals. *F/TDF* emtricitabine-tenofovir disoproxil fumarate, *HBsAg* hepatitis B surface antigen, *Anti-HBc* antibody to hepatitis B core antigen, *Anti-HBs* antibody to hepatitis B surface antigen, *CI* confidence interval, *HBV* hepatitis B virus, *HCV* hepatitis C virus

For HBV incidence, 257 initially HBV-susceptible F/TDF users were followed for a cumulative 277.78 PY, with no new HBV infections observed, resulting in an incidence rate of 0.00/100 PY (95% *CI:* 0.00–1.32). In contrast, among 109 initially HBV-susceptible F/TDF non-users followed for 130.39 PY, one new HBV case was detected, yielding an incidence rate of 0.77/100 PY (95% *CI:* 0.02–4.20; Table 2; Fig. 2).

**Table 2:** Prevalence and Incidence of HBV and HCV among F/TDF users and F/TDF non-users at enrolment, baseline and 12-month follow-up

Hepatitis	F/TDF use	Prevalence at enrolment			Prevalence at baseline			Incidence at 12-month follow-up			
		Number of cases tested	Positive cases	Prevalence (%) (95% *CI*)	Number of cases tested	Positive cases	Prevalence (%) (95% *CI*)	Number of cases tested	Number of seroconversion	PY of follow-up	Incidence per 100 PY (95% *CI*)
HBV^a^	Non-users	456	36	7.89 (5.59, 10.76)	120	0	(0.00, 3.03)	109	1	130.39	0.77 (0.02, 4.20)
	F/TDF users	1177	11	0.93 (0.47, 1.67)	259	0	(0.00, 1.41)	257	0	277.78	0.00 (0.00, 1.32)
	Daily F/TDF users	NA	NA	NA	NA	NA	NA	131	0	138.95	0.00 (0.00, 2.62)
	Event-driven F/TDF users	NA	NA	NA	NA	NA	NA	126	0	138.83	0.00 (0.00, 2.62)
HCV	Non-users	464	3	0.65 (0.13, 1.88)	461	0	(0.00, 0.80)	396	0	500.05	0.00 (0.00, 0.74)
	F/TDF users	1148	2	0.17 (0.02, 0.63)	1018	0	(0.00, 0.36)	850	3	966.57	0.31 (0.06, 0.90)
	Daily F/TDF users	NA	NA	NA	NA	NA	NA	434	1	488.14	0.20 (0.01, 1.14)
	Event-driven F/TDF users	NA	NA	NA	NA	NA	NA	416	2	478.43	0.42 (0.05, 1.50)

*F/TDF* emtricitabine-tenofovir disoproxil fumarate, *HBsAg* hepatitis B surface antigen, *Anti-HBc* antibody to hepatitis B core antigen, *Anti-HBs* antibody to hepatitis B surface antigen, *PY* person-year(s), *CI* confidence interval, *NA* Not applicable, *HBV* hepatitis B virus, *HCV* hepatitis C virus

^a^Only HBV-susceptible (HBsAg–, anti-HBc–, anti-HBs–) MSM were included at baseline and 12-month follow-up

Regarding HCV incidence, three new cases were observed among 850 initially anti-HCV-negative F/TDF users over 966.57 PY, leading to an incidence rate of 0.31/100 PY (95% *CI:* 0.06–0.90). No new HCV cases were detected among 396 initially anti-HCV-negative F/TDF non-users followed for 500.05 PY, resulting in an incidence rate of 0.00/100 PY (95% *CI:* 0.00–0.74) (Table 2).

Supplementary Material Table S3 summarises the characteristics of the one HBV-positive and three HCV-positive MSM at the 12-month follow-up. Among them, one HCV-positive individual reported more than three sexual intercourse episodes per month in the preceding three months. Additionally, one HBV-positive and two HCV-positive individuals reported CAI, with no instances of group sex reported.

### Factors associated with HBV and HCV infections at the 12-month follow-up

Table 3 presents the incidence and aIRR for HBV and HCV infections across different subgroups. F/TDF use (F/TDF vs. no F/TDF, aIRR: 0.00, 95% *CI:* 0.00–0.00; Table 3; daily F/TDF vs. no F/TDF, aIRR: 0.00, 95% *CI:* 0.00–0.00; Supplementary Material Table S4), CAI in the past three months (no vs. yes, aIRR: 0.00, 95% *CI:* 0.00–0.00), and education level (college or greater vs. high school or less, aIRR: 0.00, 95% *CI:* 0.00–0.00) were associated with a decreased HBV incidence. For HCV infections, factors including F/TDF use (no F/TDF vs. F/TDF, aIRR: 0.00, 95% *CI:* 0.00–0.25) and the number of casual male sexual partners in the past three months (none vs. > 2, aIRR: 0.00, 95% *CI:* 0.00–0.11; 1–2 vs. > 2, aIRR: 0.00, 95% *CI:* 0.00–0.11) were associated with decreased HCV incidence.

**Table 3:** Factors associated with HBV and HCV infection in the multivariate analyses (366 MSM for HBV analysis and 1246 MSM for HCV analysis at the 12-month follow-up)

Characteristic	Cases	Incidence rate (/100 PY)	aIRR^a^ (95% *CI*)
Incident HBV (*n* = 366, 408 py Follow-up, only HBV-susceptible^b^ MSM were included)			
Age, per year increase	NA	NA	0.96 (0.68, 1.32)
F/TDF			
No	1	0.77	
Yes	0	0.00	0.00 (0.00, 0.00)
CAI in the past three months			
No	0	0.00	0.00 (0.00, 0.00)
Yes	1	0.43	
Monthly frequency of sexual intercourse in the past three months			
None	1	0.98	
1–3	0	0.00	0.00 (0.00, 0.00)
> 3	0	0.00	0.00 (0.00, 0.00)
Number of casual male sexual partner in the past three months			
1–2	1	1.18	
> 2	0	0.00	0.00 (0.00, 0.00)
None	0	0.00	0.00 (0.00, 0.00)
Education level			
High school or less	1	0.77	
College or greater	0	0.00	0.00 (0.00, 0.00)
Incident HCV (*n* = 1246, 1467 py Follow-up)			
Age, per year increase	NA	NA	0.94 (0.78, 1.09)
F/TDF			
No	0	0.00	0.00 (0.00, 0.25)
Yes	3	0.31	
CAI in the past three months			
No	1	0.14	
Yes	2	0.26	1.86 (0.08, 32.14)
Monthly frequency of sexual intercourse in the past three months			
None	0	0.00	0.00 (0.00, 1.16)
1–3	2	0.33	1.50 (0.18, 52.46)
> 3	1	0.22	
Number of casual male sexual partner in the past three months			
None	0	0.00	0.00 (0.00, 0.11)
1–2	0	0.00	0.00 (0.00, 0.11)
> 2	3	0.49	
Education level			
High school or less	1	0.23	
College or greater	2	0.20	0.87 (0.02, 10.59)

*F/TDF* emtricitabine-tenofovir disoproxil fumarate, *aIRR* adjusted incidence rate ratios, *PY* person-year(s), *CAI* condomless anal intercourse, *HBsAg* hepatitis B surface antigen, *Anti-HBc* antibody to hepatitis B core antigen, *Anti-HBs* antibody to hepatitis B surface antigen, *CI* confidence interval, *HBV* hepatitis B virus, *HCV* hepatitis C virus

^a^aIRR were estimated after controlling for covariates including age, CAI in the past three months, monthly frequency of sexual intercourse in the past three months, number of casual male sexual partner in the past three months, and education level

^b^HBV-susceptible (HBsAg–, anti-HBc–, anti-HBs–)

## Discussion

To the best of our knowledge, this study is an in-depth analysis of new HBV and HCV infections among MSM receiving oral F/TDF as PrEP in China. In this real-world setting, no new HBV cases were observed among HBV-susceptible daily F/TDF users or those using event-driven F/TDF. In contrast, HBV-susceptible F/TDF non-users had a slightly higher HBV incidence rate of 0.77/100 PY. Notably, based on the aIRR analysis, HBV incidence decreased with F/TDF use. Additionally, F/TDF users had low HCV incidence rate (0.31/100 PY), although it was slightly higher than that of F/TDF non-users (0.00/100 PY). These findings may help guide HBV and HCV prevention and control strategies among MSM using F/TDF, especially in LMICs.

This study demonstrates that F/TDF is potentially effective in preventing HBV transmission among MSM, with no new cases among HBV-susceptible individuals reported during the 12-month follow-up. These results align with those of previous studies. For instance, during a 318 PY follow-up in Antwerp, Belgium, no new HBV infections were reported among 200 MSM who used daily PrEP or event-driven PrEP [26]. Similarly, a study in Tokyo, Japan, found a significantly higher annual HBV incidence among PrEP non-users (3.8/100 PY) than among daily PrEP users (0.8/100 PY) and event-driven PrEP users (no infections over 93.8 PY) [21]. Given tenofovir’s potent inhibition of HBV [27], F/TDF may offer a protective effect against HBV infection. These findings align with the World Health Organization’s goal of ending the AIDS and viral hepatitis epidemics by 2030 [28], especially considering F/TDF’s dual benefits in preventing HIV and HBV infections among high-risk populations like MSM. Moreover, broader interventions [29, 30] such as universal HBV screening, vaccination and sexual education could substantially reduce HBV transmission risks, crucial for achieving HBV elimination among MSM. However, successful outcomes depend on long-term patient retention in care and adherence monitoring, particularly due to the risk of acute liver failure in HBV-infected patients who discontinue PrEP. Further research is needed to clarify the mechanisms underlying PrEP’s protective effect against HBV and to strengthen efforts aimed at eliminating HIV and HBV as public threats among MSM by 2030.

Our HBV comprehensive testing results indicate a relatively low seropositivity rate for anti-HBs among MSM. Notably, risky sexual behaviours are more common among MSM, particularly those using PrEP, increasing their risk of HBV infection [21]. The Advisory Committee on Immunization Practices in the United States recommends universal HBV vaccination for adults aged 19–59 years [31]. Therefore, it is imperative to strengthen efforts to promote and administer HBV vaccination among MSM, particularly PrEP users, to reduce HBV incidence. Our findings indicate low self-reported HBV vaccination rates among HBV-susceptible F/TDF users (44.4%) and F/TDF non-users (44.2%). Additionally, we found a higher prevalence of actual HBV vaccine-associated immunity among F/TDF users (53.7%) compared to MSM in Beijing (38.9%) and the United States (29.5%) [32, 33]. These findings highlight a significant disparity between serologically confirmed and self-reported HBV vaccination rates among MSM in China, which may stem from the unreliability of self-reported vaccination history. The high acceptance of HBV vaccination [32] among MSM initiating PrEP suggests that healthcare providers should use PrEP consultations as an opportunity to promote HBV screening and vaccination, ensuring vaccine completion [34].

Studies have shown that HCV incidence is higher among MSM using PrEP than among those not using PrEP [7, 16]. F/TDF use may elevate HCV incidence through risk compensation mechanisms while lacking protective activity against HCV. The HCV incidence rates among F/TDF users in our study are consistent with those reported in Australia and other high-income countries [18, 35], highlighting the importance of regular STI screenings, including HCV testing, as an essential component of PrEP management.

This study aims to investigate incident HBV and HCV infections among F/TDF-using MSM in China and LMICs, offering crucial data for the prevention and control of these infections in this population. However, several limitations should be considered. First, the study was conducted in specific geographic regions in China, which may limit the generalizability of the findings to other areas. Second, self-reported data on sexual behaviour were used, which may be subject to reporting bias. Third, the potential waning effectiveness of the HBV vaccine within a decade could lead to an overestimation of its immunological impact. Therefore, healthcare providers should use PrEP consultations to promote HBV screening and vaccination [34]. Fourth, the study was a secondary analysis and did not control the sample size for direct comparisons. Despite the smaller sample size of F/TDF non-users, F/TDF use associated with lower HBV incidence rates but higher HCV incidence, potentially mediated through increased high-risk behaviors in the F/TDF users group. Further analyses with a larger population of HBV-susceptible individuals are warranted. Fifth, considering the very low number of outcome events—specifically, only one HBV incident event in the F/TDF non-users group—a larger sample size is needed to enhance the robustness of our results. Finally, unadjusted confounding factors, rather than F/TDF use itself, may explain the observed differences in HBV/HCV incidence rates between F/TDF users and non-users. Future research should address these limitations and further explore the potential impact of PrEP on HBV and HCV risk among MSM in diverse settings.

## Conclusions

This study highlights the very limited HBV and HCV incidence rates among MSM using F/TDF in LMICs. We find a low prevalence of actual HBV vaccine-associated immunity among MSM who use HIV PrEP in China. The findings suggest an association between F/TDF use and a reduced risk of HBV infection, reinforcing the benefits of F/TDF in mitigating the HBV burden among MSM. Implementing comprehensive prevention strategies—including HBV vaccination, promoting PrEP uptake and targeted health education—is crucial for effectively reducing the burden of viral hepatitis among MSM.

## Supplementary Information

Additional file 1.

## Data Availability

The raw data supporting the conclusions of this article and additional, related documents will be made available by the corresponding authors, without undue reservation.

## Abbreviations

F/TDF: Emtricitabine-tenofovir disoproxil fumarate
PrEP: Pre-exposure prophylaxis
HBV: Hepatitis B virus
HCV: Hepatitis C virus
MSM: Men who have sex with men
aIRR: Adjusted incidence rate ratios
anti-HCV: HCV antibody
LMIC: Low-income and middle-income country
STI: Sexually transmitted infection
PY: Person-years
CROPrEP: China Real-World Oral Intake of PrEP
HBsAg: Hepatitis B surface antigen
anti-HBs: Antibody to hepatitis B surface antigen
anti-HBc: Antibody to hepatitis B core antigen
CAI: Condomless anal intercourse
ELISA: Enzyme-linked immunosorbent assay
IQR: Interquartile range
*CI*: Confidence interval
