# Supplementary material for: The role of tenofovir-based HIV pre-exposure prophylaxis in preventing HBV infection among men who have sex with men: insights from China
Source: Infect Dis Poverty. 2025 Apr 27;14:31. doi: 10.1186/s40249-025-01305-9 (PMC12034112; doi:10.1186/s40249-025-01305-9)
Supplement: Supplementary file 1 — Additional file 1. [file 40249_2025_1305_MOESM1_ESM.docx]

**Supplementary Materials**

Supplementary Materials Table S1: Prevalence and Incidence of HBV among PrEP users with different hepatitis B infection status

| PrEP | Hepatitis B infection status | Prevalence at enrolment | | |  | Prevalence at baseline | | |  | Incidence at 12-month follow-up^h^ | | | |
| --- | --- | --- | --- | --- | --- | --- | --- | --- | --- | --- | --- | --- | --- |
|  |  | No. of cases tested | Positive cases | Prevalence (%)  (95% CI) |  | No. of cases tested | Positive cases | Prevalence (%)  (95% CI) |  | No. of cases tested | Number of seroconversion | PY of follow-up | Incidence per  100 PY (95% CI) |
| PrEP users | Current infection ^a^ | 5 | 5 | 100.00 (47.82, 100.00) |  | NA | NA | NA |  | NA | NA | NA | NA |
|  | Vaccine-associated immunity ^b^ | 560 | 0 | 0.00 (0.00, 0.66) |  | 525 | 0 | 0.00 (0.00, 0.70) |  | 517 | 0 | 553.6 | NA |
|  | Susceptible ^c^ | 275 | 0 | 0.00 (0.00, 1.33) |  | 259 | 0 | 0.00 (0.00, 1.41) |  | 257 | 0 | 277.8 | 0.00 (0.00, 1.32) |
|  | Immunity due to natural infection ^d^ | 193 | 0 | 0.00 (0.00, 1.89) |  | 176 | 0 | 0.00 (0.00, 2.07) |  | 174 | 0 | 198.0 | NA |
|  | Other infection status ^e^ | 19 | 0 | 0.00 (0.00, 17.65) |  | 18 | 0 | 0.00 (0.00, 18.53) |  | 18 | 0 | 19.2 | NA |
|  | Isolated HBsAg positive ^f^ | 6 | 6 | 100.00 (54.07, 100.00) |  | NA | NA | NA |  | NA | NA | NA | NA |
|  | Isolated HBsAg negative ^g^ | 119 | 0 | 0.00 (0.00, 3.05) |  | 45 | 0 | 0.00 (0.00, 7.87) |  | 43 | 0 | 28.5 | NA |
| D-PrEP | Vaccine-associated immunity ^b^ | 267 | 0 | 0.00 (0.00, 1.37) |  | 267 | 0 | 0.00 (0.00, 1.37) |  | 265 | 0 | 283.2 | NA |
|  | Susceptible ^c^ | 133 | 0 | 0.00 (0.00, 2.74) |  | 133 | 0 | 0.00 (0.00, 2.74) |  | 131 | 0 | 139.0 | 0.00 (0.00, 2.62) |
|  | Immunity due to natural infection ^d^ | 86 | 0 | 0.00 (0.00, 4.20) |  | 86 | 0 | 0.00 (0.00, 4.20) |  | 84 | 0 | 94.3 | NA |
|  | Other infection status ^e^ | 12 | 0 | 0.00 (0.00, 26.47) |  | 12 | 0 | 0.00 (0.00, 26.47) |  | 12 | 0 | 12.4 | NA |
|  | Isolated HBsAg negative ^g^ | 22 | 0 | 0.00 (0.00, 15.44) |  | 22 | 0 | 0.00 (0.00, 15.44) |  | 21 | 0 | 12.0 | NA |
| ED-PrEP | Vaccine-associated immunity ^b^ | 258 | 0 | 0.00 (0.00, 1.42) |  | 258 | 0 | 0.00 (0.00, 1.42) |  | 252 | 0 | 270.4 | NA |
|  | Susceptible ^c^ | 126 | 0 | 0.00 (0.00, 2.89) |  | 126 | 0 | 0.00 (0.00, 2.89) |  | 126 | 0 | 138.8 | 0.00 (0.00, 2.62) |
|  | Immunity due to natural infection ^d^ | 90 | 0 | 0.00 (0.00, 4.02) |  | 90 | 0 | 0.00 (0.00, 4.02) |  | 90 | 0 | 103.7 | NA |
|  | Other infection status ^e^ | 6 | 0 | 0.00 (0.00, 45.93) |  | 6 | 0 | 0.00 (0.00, 45.93) |  | 6 | 0 | 6.8 | NA |
|  | Isolated HBsAg negative ^g^ | 23 | 0 | 0.00 (0.00, 14.82) |  | 23 | 0 | 0.00 (0.00, 14.82) |  | 22 | 0 | 16.5 | NA |
| Excluded cases | Current infection ^a^ | 5 | 5 | 100.00 (47.82, 100.00) |  | NA | NA | NA |  | NA | NA | NA | NA |
|  | Vaccine-associated immunity ^b^ | 35 | 0 | 0.00 (0.00, 10.00) |  | NA | NA | NA |  | NA | NA | NA | NA |
|  | Susceptible ^c^ | 16 | 0 | 0.00 (0.00, 20.59) |  | NA | NA | NA |  | NA | NA | NA | NA |
|  | Immunity due to natural infection ^d^ | 17 | 0 | 0.00 (0.00, 19.51) |  | NA | NA | NA |  | NA | NA | NA | NA |
|  | Other infection status ^e^ | 1 | 0 | 0.00 (0.00, 97.50) |  | NA | NA | NA |  | NA | NA | NA | NA |
|  | Isolated HBsAg positive ^f^ | 6 | 6 | 100.00 (54.07, 100.00) |  | NA | NA | NA |  | NA | NA | NA | NA |
|  | Isolated HBsAg negative ^g^ | 74 | 0 | 0.00 (0.00, 4.86) |  | NA | NA | NA |  | NA | NA | NA | NA |

^a^: HBsAg+, anti-HBc+, anti-HBs-; ^b^: HBsAg-, anti-HBc-, anti-HBs+; ^c^: HBsAg-, anti-HBc-, anti-HBs-; ^d^: HBsAg-, anti-HBc+, anti-HBs+; ^e^: HBsAg-, anti-HBc+, anti-HBs-; ^f^: HBsAg+, anti-HBc untested, anti-HBs untested; ^g^: HBsAg-, anti-HBc untested, anti-HBs untested; ^h^: Only HBV-susceptible (HBsAg-, anti-HBc-, anti-HBs-) MSM were included in the calculation of HBV incidence at 12-month follow-up.

Abbreviations: HBV, Hepatitis B virus; PrEP, pre-exposure prophylaxis; HBsAg, Hepatitis B surface Antigen; anti-HBc, antibody to hepatitis B core antigen; anti-HBs, antibody to HBsAg; PY, person-year(s); CI, confidence interval; NA, Not Applicable.

Supplementary Materials Table S2: Prevalence and Incidence of HBV among PrEP non-users with different hepatitis B infection status

| Hepatitis B infection status | Prevalence at baseline | | |  | Incidence at 12-month follow-up^h^ | | | |
| --- | --- | --- | --- | --- | --- | --- | --- | --- |
|  | No. of cases tested | Positive cases | Prevalence (%) (95% CI) |  | No. of cases tested | Number of seroconversion | PY of follow-up | Incidence per  100 PY (95% CI) |
| Current infection ^a^ | 23 | 23 | 100.00 (85.18, 100.00) |  | 1 | 0 | 1.4 | NA |
| Vaccine-associated immunity ^b^ | 130 | 0 | 0.00 (0.00, 2.80) |  | 109 | 1 | 137.7 | NA |
| Susceptible ^c^ | 120 | 0 | 0.00 (0.00, 3.03) |  | 109 | 1 | 130.4 | 0.77 (0.02, 4.20) |
| Immunity due to natural infection ^d^ | 96 | 0 | 0.00 (0.00, 3.77) |  | 85 | 4 | 112.6 | NA |
| Other infection status ^e^ | 14 | 0 | 0.00 (0.00, 23.16) |  | 13 | 0 | 18.2 | NA |
| Isolated HBsAg positive ^f^ | 13 | 13 | 100.00 (75.29, 100.00) |  | NA | NA | NA | NA |
| Isolated HBsAg negative ^g^ | 60 | 0 | 0.00 (0.00, 5.96) |  | 42 | 0 | 55.7 | NA |

^a^: HBsAg+, anti-HBc+, anti-HBs-; ^b^: HBsAg-, anti-HBc-, anti-HBs+; ^c^: HBsAg-, anti-HBc-, anti-HBs-; ^d^: HBsAg-, anti-HBc+, anti-HBs+; ^e^: HBsAg-, anti-HBc+, anti-HBs-; ^f^: HBsAg+, anti-HBc untested, anti-HBs untested; ^g^: HBsAg-, anti-HBc untested, anti-HBs untested; ^h^: Only HBV-susceptible (HBsAg-, anti-HBc-, anti-HBs-) MSM were included in the calculation of HBV incidence at 12-month follow-up.

Abbreviations: HBV, Hepatitis B virus; PrEP, pre-exposure prophylaxis; HBsAg, Hepatitis B surface Antigen; anti-HBc, antibody to hepatitis B core antigen; anti-HBs, antibody to HBsAg; PY, person-year(s); CI, confidence interval; NA, Not Applicable.

Supplementary Materials Table S3: Characteristics of HBV and HCV positive participants among MSM in CROPrEP

| Case | HBV+/HCV+ | Diagnosed time point | PrEP | Age (y) | Site | Monthly frequency of sexual intercourse^*^ | Sexual role with man^*^ | No. of regular sexual partner^*^ | No. of casual sexual partner^*^ | CAI^*^ | Group sex^*^ |
| --- | --- | --- | --- | --- | --- | --- | --- | --- | --- | --- | --- |
| 1 | HBV+ | M12 | PrEP Nonuser | 38 | Shenyang | <1 | Versatile | <1 | 1-2 | Yes | No |
| 2 | HCV+ | M12 | D-PrEP | 34 | Beijing | >3 | Bottom | >2 | 1-2 | Yes | No |
| 3 | HCV+ | M12 | ED-PrEP | 37 | Shenyang | 1-3 | Versatile | <1 | 1-2 | No | No |
| 4 | HCV+ | M12 | ED-PrEP | 35 | Beijing | 1-3 | Versatile | >2 | 1-2 | Yes | No |

^*^: in the past three months. Abbreviations: M0, enrolment time point; M12, 12-month follow-up time point; D-PrEP, daily pre-exposure prophylaxis; ED-PrEP, event-driven pre-exposure prophylaxis; CAI, condomless anal intercourse.

Supplementary Materials Table S4: Factors associated with HBV infection in the multivariate analyses (subgroup analysis of event-driven and daily F/TDF).

| Characteristic | Cases | Incidence Rate (/100PY) | | aIRR^a^ (95% CI) |
| --- | --- | --- | --- | --- |
| **Incident HBV (n=366, 408 py Follow-up, only HBV-susceptible^b^ MSM were included)** | | | | |
| Age, per year increase | NA | | NA | 0.96 (0.73, 1.25) |
| Event-driven or daily F/TDF |  | |  |  |
| No-user | 1 | | 0.77 |  |
| Event-driven F/TDF | 0 | | 0.00 | 0.00 (0.00, +∞) |
| Daily F/TDF | 0 | | 0.00 | 0.00 (0.00, 0.00) |
| CAI in the past three months |  | |  |  |
| No | 0 | | 0.00 | 0.00 (0.00, 0.00) |
| Yes | 1 | | 0.43 |  |
| Monthly frequency of sexual intercourse in the past three months | | | |  |
| None | 1 | | 0.98 |  |
| 1-3 | 0 | | 0.00 | 0.00 (0.00, 0.00) |
| >3 | 0 | | 0.00 | 0.00 (0.00, 0.00) |
| Number of casual male sexual partner in the past three months | | | |  |
| 1-2 | 1 | | 1.18 |  |
| >2 | 0 | | 0.00 | 0.00 (0.00, 0.00) |
| None | 0 | | 0.00 | 0.00 (0.00, 0.00) |
| Education level |  | |  |  |
| High school or less | 1 | | 0.77 |  |
| College and greater | 0 | | 0.00 | 0.00 (0.00, 0.00) |

aIRR: adjusted incidence rate ratio; F/TDF: emtricitabine–tenofovir disoproxil fumarate; CAI: condomless anal intercourse; ^a^: adjusted for the listed variables; ^b^: HBV-susceptible (HBsAg-, anti-HBc-, anti-HBs-).

STROBE Statement—Checklist of items that should be included in reports of ***cohort studies***

|  | Item No | Recommendation | Page  No. | Relevant text from manuscript |
| --- | --- | --- | --- | --- |
| **Title and abstract** | 1 | (*a*) Indicate the study’s design with a commonly used term in the title or the abstract | 2 | multicentre, prospective study |
|  |  | (*b*) Provide in the abstract an informative and balanced summary of what was done and what was found | 2 | Abstract |
| Introduction | | |  |  |
| Background/rationale | 2 | Explain the scientific background and rationale for the investigation being reported | 3-4 | Introduction, paragraph 1-3 |
| Objectives | 3 | State specific objectives, including any prespecified hypotheses | 4 | Introduction, paragraph 4 |
| Methods | | |  |  |
| Study design | 4 | Present key elements of study design early in the paper | 4-5 | Methods, Study design and population, paragraph 1 |
| Setting | 5 | Describe the setting, locations, and relevant dates, including periods of recruitment, exposure, follow-up, and data collection | 4-5 | Methods, Study design and population, paragraph 1-2 |
| Participants | 6 | (*a*) Give the eligibility criteria, and the sources and methods of selection of participants. Describe methods of follow-up | 4-5 | Methods, Study design and population, paragraph 1-2 |
|  |  | (*b*) For matched studies, give matching criteria and number of exposed and unexposed | NA | NA |
| Variables | 7 | Clearly define all outcomes, exposures, predictors, potential confounders, and effect modifiers. Give diagnostic criteria, if applicable | 5-6 | Methods, Outcomes; Methods, Laboratory procedures |
| Data sources/ measurement | 8* | For each variable of interest, give sources of data and details of methods of assessment (measurement). Describe comparability of assessment methods if there is more than one group | NA | NA |
| Bias | 9 | Describe any efforts to address potential sources of bias | 6 | Methods, Statistical Analysis, paragraph 1 |
| Study size | 10 | Explain how the study size was arrived at | 6 | Methods, Statistical Analysis, paragraph 1 |
| Quantitative variables | 11 | Explain how quantitative variables were handled in the analyses. If applicable, describe which groupings were chosen and why | 6 | Methods, Statistical Analysis, paragraph 1 |
| Statistical methods | 12 | (*a*) Describe all statistical methods, including those used to control for confounding | 6 | Methods, Statistical Analysis, paragraph 2 |
|  |  | (*b*) Describe any methods used to examine subgroups and interactions | 6 | Methods, Statistical Analysis, paragraph 2 |
|  |  | (*c*) Explain how missing data were addressed | 6 | Methods, Statistical Analysis, paragraph 2 |
|  |  | (*d*) If applicable, explain how loss to follow-up was addressed | NA | NA |
|  |  | (*e*) Describe any sensitivity analyses | NA | NA |
| Results | | |  |  |
| Participants | 13* | (a) Report numbers of individuals at each stage of study—eg numbers potentially eligible, examined for eligibility, confirmed eligible, included in the study, completing follow-up, and analysed | 7 | Results, Enrolment and characteristics of study participants, paragraph 1 |
|  |  | (b) Give reasons for non-participation at each stage | 17 | Figure 1 |
|  |  | (c) Consider use of a flow diagram | 17 | Figure 1 |
| Descriptive data | 14* | (a) Give characteristics of study participants (eg demographic, clinical, social) and information on exposures and potential confounders | 7 | Results, Enrolment and characteristics of study participants, paragraph 2-3 |
|  |  | (b) Indicate number of participants with missing data for each variable of interest | NA | NA |
|  |  | (c) Summarise follow-up time (eg, average and total amount) | 20 | Table 2 |
| Outcome data | 15* | Report numbers of outcome events or summary measures over time | 20 | Table 2 |
| Main results | 16 | (*a*) Give unadjusted estimates and, if applicable, confounder-adjusted estimates and their precision (eg, 95% confidence interval). Make clear which confounders were adjusted for and why they were included | 21 | Figure 2 |
|  |  | (*b*) Report category boundaries when continuous variables were categorized | 18-21 | Tables 1-2; Figures 1-2 |
|  |  | (*c*) If relevant, consider translating estimates of relative risk into absolute risk for a meaningful time period | NA | NA |
| Other analyses | 17 | Report other analyses done—eg analyses of subgroups and interactions, and sensitivity analyses | NA | NA |
| Discussion | | |  |  |
| Key results | 18 | Summarise key results with reference to study objectives | 9 | Discussion, paragraph 1 |
| Limitations | 19 | Discuss limitations of the study, taking into account sources of potential bias or imprecision. Discuss both direction and magnitude of any potential bias | 11-12 | Discussion, paragraph 7 |
| Interpretation | 20 | Give a cautious overall interpretation of results considering objectives, limitations, multiplicity of analyses, results from similar studies, and other relevant evidence | 9-12 | Discussion, paragraph 2-6 |
| Generalisability | 21 | Discuss the generalisability (external validity) of the study results | 12 | Discussion, paragraph 8 |
| Other information | | |  |  |
| Funding | 22 | Give the source of funding and the role of the funders for the present study and, if applicable, for the original study on which the present article is based | NA | NA |

*Give information separately for exposed and unexposed groups.

**Note:** An Explanation and Elaboration article discusses each checklist item and gives methodological background and published examples of transparent reporting. The STROBE checklist is best used in conjunction with this article (freely available on the Web sites of PLoS Medicine at http://www.plosmedicine.org/, Annals of Internal Medicine at http://www.annals.org/, and Epidemiology at http://www.epidem.com/). Information on the STROBE Initiative is available at http://www.strobe-statement.org.
